# Effect of psychological first aid program on stress level and psychological well-being among caregivers of older adults with alzheimer’s disease

**DOI:** 10.1186/s12912-022-01049-z

**Published:** 2022-10-10

**Authors:** Eman Mahmoud Mohammed Shoukr, Abeer Abd El-Rahman Mohamed, Ayman Mohamed El-Ashry, Heba Ahmed Mohsen

**Affiliations:** Faculty of Nursing, Alexandria University, Alexandria, Egypt

**Keywords:** Psychological first aid, Older adults, Alzheimer’s disease, Caregiver stress, Psychological well-being.

## Abstract

**Introduction:**

Older adults with Alzheimer’s disease (AD) experience drastic changes in their physical and mental abilities. AD patients became heavily dependent on their caregivers for everyday functions, which have significant implications not only for them but also for their caregivers. So, many AD caregivers experienced an increased level of depression and anxiety symptoms, lower perceived control, and higher burden compared to non-AD caregivers. Therefore, psychological first aid (PFA) and educational interventions are designed to enable those caregivers to meet the daily requirements of their patient care and to cope with its challenges.

**Aim:**

Determine the effect of psychological first aid program on stress level and psychological well-being among caregivers of older adults with Alzheimer’s disease.

**Design:**

One group pre-test post-test was followed.

**Subjects:**

A convenience sample of one hundred (100) caregivers of older adults with AD.

**Setting:**

All online groups concerned with the care of Alzheimer’s disease patients on Facebook.

**Tools:**

Socio-demographic and clinical data of older adults with Alzheimer’s disease and their caregivers’ questionnaire, Alzheimer’s disease knowledge scale, Kingston caregiver stress scale, and authentic identity measures (AIM) scale of psychological well-being

**Results:**

The psychological first aid program has highly statistically significant effect on the AD caregivers’ knowledge, stress level and psychological well-being as (t=-30.707, P = 0.000, t = 8.500, P = 0.000 & t= -4.763, P = 0.000 respectively).

**Conclusion:**

Psychological first aid program is considered an effective intervention in decreasing the AD caregivers’ stress and increasing their psychological wellbeing and knowledge regarding delivering care for AD patients.

## Introduction

The caregiver is crucial in the lives of those who receive care, either formal or informal caregivers. Formal caregivers are trained professionals who assist elderly patients [1]. Informal caregivers are frequently friends or family members who provide long-term care for an elderly patient [2]. The majority of informal caregivers are females and reside in the same household as the person they are caring for [3]. A family caregiver is responsible for providing and coordinating care for an older adult parent who needs long-term care [4]. Caring for an older person with AD is more difficult than caring for a loved one with other chronic diseases or impairments [5]. Dementia caregivers are more likely than nondementia caregivers to provide more hours of care, to assist elders with personal activities of daily living (ADLs), to protect them from harm, to deal with agitated behaviors and to provide more types of long-term care [6–8]. These responsibilities tire caregivers and make them vulnerable to stress-related disorders [9, 10]. As the number of elderly individuals who have AD increases, the amount and severity of the burden associated with the disease are expected to rise [11]. According to the Alzheimer’s Association, the number of people aged 65 and above with AD is expected to increase from 5.1 million in 2015 to 7.1 million by 2025. By 2050, the number of people in the world will double [12].

Alzheimer’s disease (AD) is a severe disease that slowly erodes an elderly person’s memory and capacity to think, make decisions, communicate, and carry out daily tasks. As Alzheimer’s disease advances, older adults may notice changes in their personality and conduct. Once symptoms of Alzheimer’s appear, people can expect to live for 8 to 12 years [13]. Individuals suffering from this degenerative brain disease appear to go through a variety of stages, ranging from mild cognitive impairment to severe cognitive impairment [14]. As the condition progresses, the patient loses independence and becomes increasingly reliant on others for day-to-day tasks. These extreme changes in physical and mental capacities have far-reaching ramifications not only for diseased loved ones but also for caregivers [15, 16].

AD caregivers may be more affected by a variety of emotional and physical stressors than non-AD caregivers. Unpaid family caregivers face high levels of emotional stress, despair, and anxiety [17]. The prevalence of depression rates in Alzheimer’s caregivers ranged from 14 to 81% [18, 19]. Burdens can impair caregivers’ abilities and take several types, such as perceived burdens, subjective burdens, or objective burdens [20]. The socio-demographic status of the caregiver and the patient, the nature of the patient’s disease, the duration and severity of dementia, the educational level of elderly patients, cultural influences, and the perceived stress arising from caregiving are all factors linked to caregiver stress [21]. Despite the negative physical and mental impact of caregiving, the caregiver-patient relationship is critical in improving the quality of life for both the patient with AD and the caregiver. Caregivers may need a way to release their tension and anxiety [22, 23].

Psychological wellbeing (PWB) is described as a person’s level of psychological happiness/health, which includes life satisfaction and accomplishment sentiments. The psychological well-being of caregivers who have patients suffering from AD is directly affected by four factors: burden, perceived social support, hours of caregiving, and self-esteem [24]. Therefore, these factors should be investigated and involved in the caring process to improve caregivers’ psychological well-being over time [25–27]. Psychological first aid (PFA) has been chosen as the primary strategy for psychological rehabilitation after crisis. This may be a sign of the strong need to feel informed and in control while working with troubled family caregivers; to know what to do and how to best respond in order to decrease suffering and encourage healing [28, 29]. Assess people’s needs and worries, and assist them in meeting their basic needs, such as rest, sleep, exercise, and consuming healthy food. Additionally, listening without pressuring them, soothing them, connecting them to required information and services, social support by joining support groups, practicing relaxation techniques, engaging in positive distracting events such as sports, interests, reading, trying to sustain a normal schedule to the level possible and safeguarding them from further damage are all parts of PFA [30–32].

The RAPID model is a method for providing PFA. The RAPID methodology focuses on building **rapport** by accompanying gerontological nurses as active listeners. **Assessing needs** in determining whether the person being cared for is distressed or dysfunctional (unable to do what is required). Then, immediate care should be prioritized by focusing on the ability to meet immediate needs as well as identifying people in higher-risk situations. After that **intervening** (stabilizing if the person is not stable, then mitigating immediate challenges by coming alongside, providing support, normalizing, educating where necessary, and connecting the person with resources). Finally, the concept of **disposition** determines whether the person has regained the functional capacity to engage in basic daily living activities [33, 34]. The RAPID model concepts imply that PFA focuses on urgent needs, anticipates the need for additional care, and seeks it [35, 36].

PFA provides emotional and practical support to caregivers, families, or communities who have difficulty coping. It is about making a connection with people in a compassionate, nonjudgmental manner to bring calm and comfort [37]. PFA uses the RAPID model to reduce the stigma associated with mental health crises and negative health outcomes in the general public as well as community-building strategies on self-care and promoting conversations about wellness [38]. PFA has three key concepts: **look** (for safety or for individuals who require assistance), **listen** (to those who are distressed), and **link** (to further support) [39]. Understanding and taking care of caregivers of older adults with AD is a critical part of providing proper care for Alzheimer’s patients because it is difficult to support the patients if caregivers are not taking care of themselves. Caregivers’ self-care plans involve identifying their support systems and protective factors that will be used to manage stress to maintain their physical, mental, and emotional health [40].

The gerontological nurse has an essential role in psycho-educational support groups, which are included in PFA and relate to the practice of employing psychological and educational principles to assist people in learning and maturing. The gerontological nurse should emphasize the cognitive and affective aspects of learning [41]. The aims of psychoeducational and counseling therapies, which are performed by gerontological nurses, are to improve caregivers’ knowledge, skills, and confidence. As well as creating trustworthy and accessible networks of support for them. In addition, it improves caregivers’ psychological well-being and delays the institutionalization of the care recipient [20]. Moreover, PFA boosts caregivers’ psychological resilience, enables greater support, even in virtual form or through telecommunications, and improves the well-being of AD patients’ caregivers [42]. Furthermore, the nurse teaches caregivers stress-reduction techniques and increases their knowledge of coping skills, allowing them to reframe and reduce the stress of caregiving as well as provide resources and information on managing their health [9].

**In this context, this study aimed to** determine the effect of a PFA program on stress levels and psychological well-being among caregivers of older adults with Alzheimer’s disease.

### Research Hypotheses

It was hypothesized that.

- The caregivers of older adults with Alzheimer’s disease who receive the PFA program exhibit lower stress levels after the application of the study intervention program than before it.
- The caregivers of older adults with Alzheimer’s disease who receive the PFA program exhibit higher psychological well-being levels after the application of the study intervention program than before it.

## Materials

### Design

The study followed a one-group pretest-posttest design.

### Setting

Based on the specifications concerning the restrictions during the COVID-19 outbreak in the country, the researchers chose the online platform for data collection. The study subjects were the caregivers who provided care to older adults with Alzheimer’s disease. The researchers surveyed all groups concerned with the care of Alzheimer’s disease patients on Facebook, which is a social media platform, and selected the subjects from these groups. These groups were as follows: Alzheimer’s disease friends, Alzheimer’s in Arabic, Alzheimer’s patients’ friends, Alzheimer’s patients in the Arab world, Support for families of Alzheimer’s patients, Alzheimer’s disease, My experience with Alzheimer’s mom, and me, A beacon of hope for Alzheimer’s patients, and Fighting Alzheimer’s in the Arab world.

### Subjects: Sample size calculation and sampling technique

A convenience sample of one hundred (100) caregivers of older adults with AD selected from the abovementioned groups was included in this study. The participants were estimated using the G*Power Windows 3.1.9.7 program with the following parameters: effect size = 0.5, α err prob = 0.05, power (1-β err prob) = 0.95. The program revealed a minimum sample size of 54 older adults. The sample size was increased to 100 during the data collection to consider 10% non-responses. The inclusion criteria were as follows: aged 18 years and above, both sexes, able to read and write, act as a caregiver to older adults with Alzheimer’s disease, willing to participate in the study, and have access to the internet via any method such as smartphones, laptops, or tablets.

### Four tools were used to collect the necessary data as follows

**Tool (I): Sociodemographic and Clinical Data of Older Adults with Alzheimer’s Disease and their Caregivers Questionnaire**: This tool was established by the researchers based on related literature to collect the sociodemographic and clinical data of older adults with Alzheimer’s disease and their caregivers. This tool included two parts as follows:

**Part one** Socio-demographic and clinical data of caregivers of older adults with Alzheimer’s disease. It included items such as age, sex, marital status, educational level, residence, working status, income, medical history, diagnosis, and pharmacological treatment. Items about the duration of caregiving, numbers of hours spent in caregiving, and the presence of others helping in the caregiving process.

**Part two** Socio-demographic and clinical data of older adults with Alzheimer’s disease. It included items such as age, sex, level of activity and mobility status, duration of being diagnosed with AD, other chronic illnesses, and pharmacological treatment.

### Tool (II): Alzheimer Disease Knowledge Scale (ADKS)

This tool was established by **Carpenter et al., 2009** to assess the current general knowledge about AD. It is a simple, short, and reliable scale, as the test-retest reliability coefficient was 0.81, and its p < 0.001, with adequate psychometric properties designed for use in both applied and research contexts and capable of assessing knowledge about AD among the public, patients, caregivers, and professionals. The scale comprises 30 items having true or false answers taking approximately 5–10 min to complete, with the resulting score being the number answered correctly, with a total score ranging from 0 to 30. However, the ADKS is conceptually split into seven subscales: life impact, risk factors, symptoms, treatment & management, assessment & diagnosis, caregiving, and course of the disease. It is best expressed by the overall knowledge score rather than separately scored subscales to indicate the level of general knowledge about AD and the test–retest reliability coefficient was r = 0.81 which suggesting adequate test–retest reliability [43]. The reliability test in the present study reflected good reliability (r = 0.822). In addition, the researchers conducted an exploratory factor analysis to test the validity of this tool, the loading ranged from 0.411 to 0.977 before rotation while it ranged from 0.524 to 0.932 after varimax rotation which is greater than 0.35 and accounting for 78.151% of the total variance. Moreover, the Kaiser-Meyer-Olkin measure of sampling adequacy was 0.903, which suggests that these data were very suitable for factor analysis as well as Bartlett’s test of sphericity reached statistical significance (P = 0.000), which supported the factor ability of the correlation matrix, so the items of this scale were retained.

### Tool (III): Kingston Caregiver stress scale (KCSS)

This tool was established by **Hopkins and Kilik in 2016** to quickly (in fewer than 5 min) allow a caregiver to express the amount of stress that he/she is feeling. The KCSS divides caregiver stress into a more comprehensive set of ten questions that represent different potential sources of stress to the lay caregiver: care-related feelings, family matters, and any financial stress. For each question, the degree of stress was rated on a 1 to 5 anchored scale, ranging from (1) feeling fine/no stress (coping fine/no problems), (2) some stress, (3) moderate stress, (4) a lot of stress, to (5) extreme stress (feeling “at the end of the rope”, health at risk). The total score ranged from 10 to 50. The analysis of this tool showed satisfactory reliability, with Cronbach’s alpha = 0.85 [44]. This tool was tested for its reliability and the results indicated that it is reliable (r = 0.891). In addition, the researchers conducted an exploratory factor analysis to test the validity of this tool, and the loading ranged from 0.713 to 0.816 before rotation while it ranged from 0.563 to 0.882 after varimax rotation which is greater than 0.35 and accounting for 67.547% of the total variance. Moreover, the Kaiser-Meyer-Olkin measure of sampling adequacy was 0.895, which suggests that these data were very suitable for factor analysis as well as Bartlett’s test of sphericity reached statistical significance (P = 0.000), which supported the factor ability of the correlation matrix so, the items of this scale were retained.

### Tool (IV): AutauthenticeidentityameasuresIM) Scale of Psychological Well-Being

This scale was developed by **Petersen in 2018** to assess psychological well-being. It consists of nine cognitive domains: purpose, optimism, self-esteem, autonomy, self-efficacy, relatedness, competence, other-esteem, and other-efficacy. Each domain was represented by a single item with a six-point Likert scale. Scores for each item were then aggregated, providing a possible range of 9 (all strongly disagree) to 54 (all strongly agree). A high score represents someone with positive psychological strengths. The internal reliability for the AIM scale was high, as Cronbach’s alpha was 0.87 [45]. This tool was translated into the Arabic language by the researchers and tested for its reliability, and the results indicated that it is reliable with a Cronbach’s alpha of 0.784. In addition, the researchers conducted an exploratory factor analysis to test the validity of this tool, and the loading ranged from 0.442 to 0.829 before rotation while it ranged from 0.580 to 0.953 after varimax rotation which is greater than 0.35 and accounting for 68.606% of the total variance. Moreover, the Kaiser-Meyer-Olkin measure of sampling adequacy was 0.943, which suggests that these data were very suitable for factor analysis as well as Bartlett’s test of sphericity reached statistical significance (P = 0.000), which supported the factor ability of the correlation matrix so, the items of this scale were retained.

### Method

The study was carried out through three phases: a **preparation phase, implementation phase, and evaluation phase.** In **the preparation phase**, the necessary approval and permission to conduct the study were obtained from the Research Ethics Committee, Faculty of Nursing, Alexandria University. Moreover, tools I-IV were translated into the Arabic language by the researchers to be revised and tested for their validity by a panel of seven specialists in the related fields: gerontological nursing, psychiatric nursing, and community health nursing. The Lawshe Content Validity Ratio was 1 and higher than 0.99, which indicates the content validity of the tools. The tools were assessed for their comprehensiveness, clarity, relevance, and applicability. Then, the study tools were prepared and formulated on the Google Form, which facilitated the online sharing of the questionnaire and enabled the researchers to maintain the confidentiality of the study subject’s data. The researchers tested the tool link to determine if it renders correctly in various browsers.

Moreover, the researchers surveyed all groups concerned with Alzheimer’s disease on the Facebook platform and informed the caregivers of older adults with Alzheimer’s disease on these groups about the purpose of the study as well as the date and time of data collection. Then, the caregivers who agreed to participate in the study were asked to send their phone numbers to the researchers on the messenger to be added to the group created by the researchers on WhatsApp. The researchers named the WhatsApp group “Caring with Alzheimer Patients”.

Informed consent was required from all study subjects who fulfilled the criteria before including them in the study, so it was sent to them, and they were asked to print the consent sign their names, scan or take a photo of the consent and resend it to the researchers through WhatsApp group or by email. The study subjects, who did not master the skill to print, scan, or take a photo of the consent, had the choice to read the consent and record their approval and to send it at WhatsApp or messenger. Additionally, a pilot study was performed on 20 caregivers who were not included in the study to assess the applicability, clarity, and feasibility of the study tools. Then, the necessary modifications were made accordingly.

Furthermore, the researchers assessed the needs of the study subjects by meeting them online through the Zoom application and asking them about their needs in the caregiving process as well as asking them an open-ended question about their needs in the Google form. Then, the proposed nursing intervention program was prepared by the researchers based on the study subjects’ needs [4, 14, 16]. The program was planned to be carried out in 10 sessions that were classified into sessions regarding knowledge about AD in older adults and how to care for them and sessions regarding caring for the caregivers of older adults with AD, as shown in Table 1.

**Table 1:** Description and illustration of objective, duration, and content of psychological first aid program

Sessions	Objective	Duration	Content
A. Sessions regarding knowledge about the AD in older adults and how to care for them			
1st session	- Increase the caregivers’ awareness of Alzheimer’s disease and its stages.	60 min	- History of Alzheimer’s disease - Definition of Alzheimer’s disease - Risk factors for Alzheimer’s disease - Signs and symptoms of Alzheimer’s disease - Stages of Alzheimer’s disease - Treatment of Alzheimer’s disease
2nd session	- Identify common behavioral problems encountered by older adults with Alzheimer’s disease and how to deal with these behaviors.	60 min	- Common changes in behaviors of older adults with Alzheimer’s disease. - Triggering situations of these behaviors - Factors contributing to the development of these behaviors. - Different behavioral problems encountered by older adults with Alzheimer’s disease as aggression, anger, anxiety, agitation, and general emotional distress. - Coping tips to deal with these behaviors.
3rd session	- Identify common behavioral problems encountered by older adults with Alzheimer’s disease and how to deal with these behaviors.	60 min	- Continuing the different behavioral problems encountered by older adults with Alzheimer’s disease as restlessness, pacing, shredding of paper or tissues, hallucinations, delusions, physical or verbal outbursts, sleep issues, and sundowning. - Coping tips to deal with these behaviors.
4th session	- Identify common nutritional problems encountered by older adults with Alzheimer’s disease. - Identify tips for good nutrition for Alzheimer’s older adults. - Maintain the oral health of Alzheimer’s older adults.	60 min	- Nutritional problems encountered by older adults with Alzheimer’s disease. - Nutrition tips for older adults with Alzheimer’s disease. - How the caregivers can make mealtimes easier for older adults with Alzheimer’s disease. - Promoting independence of the older adults with Alzheimer’s disease during the meal. - Minimizing eating and nutrition problems for older adults with Alzheimer’s disease. - Maintaining oral health of the older adults with Alzheimer’s disease.
5th session	- Identify how to communicate with Alzheimer’s older adults. - Identify how to care for Alzheimer’s older adults with urinary incontinence. - Perform bed baths for bedridden Alzheimer’s older adults. - Maintain the skin integrity of Alzheimer’s older adults.	60 min	- Communication with Alzheimer’s older adults: ♣ Changes that occur in communication with Alzheimer’s geriatric patient ♣ How to communicate with Alzheimer’s older adults in the early stage, middle stage, or late stage. - Daily care of Alzheimer’s older adults and how to organize the daily activities. - Care for Alzheimer’s older adults with urinary incontinence. - How to care for Alzheimer’ older adults’ skin and how to maintain their skin integrity.
6th session	- Identify how to care for Alzheimer’s older adults in the early, middle, and late stages.	60 min	- Caring for early-stage Alzheimer’s older adults. - Caring for middle-stage Alzheimer’s older adults. - Caring for late-stage Alzheimer’s older adults. - The caregiving responsibilities of the Alzheimer’s older adult’s caregiver in the early, middle, and late stages.
B. Sessions concerned with caring for Alzheimer’s older adults’ caregivers			
7th session	- Enrich the Alzheimer older adults’ caregivers with the required knowledge and skills to deal with the caregiving stressors and stress.	60 min	- Signs and symptoms of Alzheimer older adults’ caregiver stress. - General advice that helps Alzheimer older adults’ caregivers to adapt to their caregiving roles. - Strategies to deal with the caregiving stress - Using relaxation techniques: ♣ Visualization (imagine a place or situation that is peaceful and calm) ♣ Meditation (dedicating 15 min a day to decrease all stressful thoughts) ♣ Deep breathing exercises ♣ Progressive muscle relaxation
8th session	- Decrease the Alzheimer’s older adults’ caregivers’ stress through practicing exercises.	60 min	- Teach the Alzheimer’s older adults’ caregivers how to care for their health through practicing exercises by teaching them the following: ♣ Importance of exercise ♣ Principles of practicing exercises ♣ Examples of exercises they can do and how to perform them.
9th session	- Teach the Alzheimer’s older adults’ caregivers how to enhance their sleep patterns.	60 min	- Sleep problems encountered by Alzheimer’s older adults’ caregivers: ♣ Sleep problems, causes, and its impact. ♣ Importance of sleep ♣ Strategies to enhance caregivers’ sleep pattern
10th session	- Maintain the nutritional status of the Alzheimer older adults’ caregivers.	60 min	- Tips for enhancing the nutritional status of the caregivers of older adults with Alzheimer’s disease. ♣ Importance of good nutrition ♣ Good nutrients. ♣ Tips of healthy food preparation and storage.

### Implementation phase

**Fig. 1:**
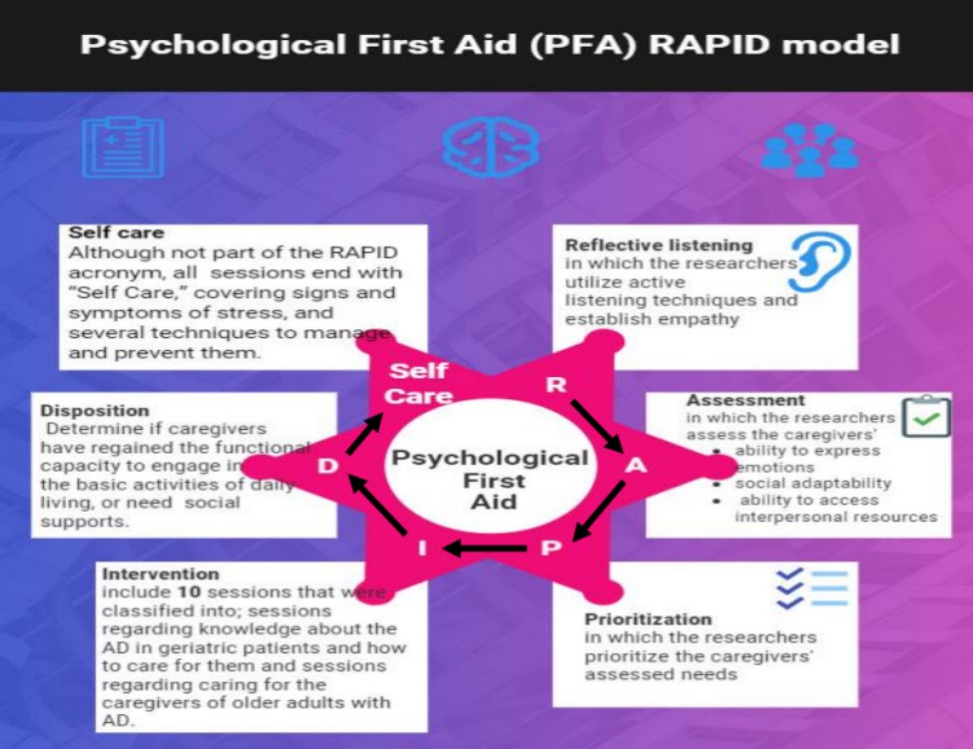
Represents the Johns Hopkins RAPID model of psychological first aid [42] Note: In this study, *the researchers designed this figure for easy presentation of the study program that was developed based on the RAPID model as* follows **R** refers to reflective listening to the Alzheimer patients’ caregivers, **A** indicates assessment of their needs, **P** reflects the prioritization of these needs, **I** for intervention that was performed in this study and **D** reflects disposition in which there was a determination of the caregivers’ functional capacity

The researchers sent the Google Form link of the tools to the study subjects in the WhatsApp group, and they were asked to fill it in as a pretest to assess the necessary data before the application of the study intervention program. The responses were stored in a worksheet that could only be accessed through a Google account login. Then, the researcher carried out the study intervention program by following the Johns Hopkins model of PFA (RAPID—PFA) [46, 47]. The RAPID acronym stands for Reflective listening, Assessment of needs, Prioritization, Intervention, and Disposition. In this study, the researchers designed Fig. 1 for easy presentation of the study program that was developed based on the RAPID model as follows: **R** refers to reflective listening to the Alzheimer patients’ caregivers, **A** indicates assessment of their needs, **P** reflects the prioritization of these needs, **I** for intervention that was performed in this study and **D** reflects disposition in which there was a determination of the caregivers’ functional capacity.

The model steps were implemented as follows: first, the researchers instructed the study subjects to download the ZOOM meeting application on their smartphones after illustrating the steps of downloading it to them and scheduled several meetings to actively listen to the caregivers to identify their problems and stressors. The caregivers who did not install the ZOOM meetings program were asked about the preferred way to communicate their problems to the researchers either on their private WhatsApp or messenger or phone. Moreover, the researchers assessed the caregivers’ needs in the caregiving process, then prioritized their needs, and then implemented the study intervention program.

The researchers prepared an illustrative PowerPoint presentation of each study program session and scheduled a ZOOM meeting at a suitable time for the study subjects to illustrate each session and answer the study subjects’ questions. Moreover, the researchers recorded the sessions based on the study subjects’ requests. In addition, the study subjects asked the researchers to make a YouTube channel to upload the recorded video of each session to be available to view at any time, and the researchers met their request. The link of the channel is as follows: https://youtube.com/channel/UCDqJUy6l46QBceNpNvscOjg. In addition, the researchers shared the link of each recorded video on the WhatsApp groups after the completion of each session and answered the study subjects’ questions.

Furthermore, the researchers made reminiscence therapy for the study subjects by asking them to share their experiences of caregiving and acting as a support group for each other. Additionally, the researchers were available to answer the study subjects’ questions and inquiries all the time in WhatsApp.

### Evaluation phase

After the implementation of the study interventions, the researcher resent the posttest by sending the Google form link to assess the caregivers’ knowledge about Alzheimer’s disease, stress level, and psychological wellbeing immediately after the implementation of the study intervention program using tools II-IV. The data collection process started from the beginning of July 2020 until the end of February 2021. The proposed interventions were evaluated by using proper statistical analysis.

### Ethical considerations

The necessary formal approval and permission to conduct the study were obtained from the Research Ethics Committee of the Faculty of Nursing, Alexandria University, Egypt. Informed written and verbal consent were obtained from each study subject included in this study after an explanation of the study purpose. Study subjects’ privacy and anonymity were maintained along with the confidentiality of the collected data. The researchers informed the study subjects that they had the right to withdraw from the study at any time.

### Statistical Analysis

After data were collected, they were revised, coded, and input into the statistical software IBM SPSS version 26. The reliability of the tools was determined by Cronbach’s alpha. Frequency tables and cross-tabulation were used to illustrate the results. Quantitative data were summarized by the arithmetic mean, standard deviation, and mean score percent. All statistical analyses were performed using two-tailed tests and an alpha error of 0.05. A P-value less than or equal to 0.05 was considered statistically significant. Descriptive statistical analysis included the mean with standard deviation, minimum and maximum for the numeric data and percent to describe the frequency of each category for categorical data. Inferential statistical analysis included paired sample t-test: This parametric statistical test is used to compare the mean scores for numeric variables between two measures for the same group if the variable follows the normal distribution.

## Results

Table 2 indicates that the mean age of the studied caregivers was 41.57 ± 2.78 years (range 21 to 57 years). The table shows that 41% of the studied caregivers were between 40 and 50 years of age, and the majority of the studied caregivers were females (83%). Concerning marital status, 63% of the studied caregivers were married. Regarding the education level, 51% of the studied caregivers had a university education, followed by 20% of them having a postgraduate education. Regarding the relationship of the caregivers to their care receiver, their daughters constituted 73% of the caregivers, while 16% of their sons were caregivers. Additionally, 67% of the caregivers lived with their Alzheimer’s disease patients. Approximately half of the studied caregivers had children under the age of 18. Regarding work in addition to caregiving, 36% of them were employees. Regarding income, 61% of the studied caregivers reported having enough income. Concerning the number of hours spent in caregiving, 29% of the studied caregivers reported spending less than 6 h in caregiving, and only 4% reported spending 18–24 h in caregiving. In addition, more than half 59% of the studied caregivers reported providing care to their Alzheimer’s patients for more than two years. Finally, 52% of the studied caregivers reported having chronic diseases, and 55% of them took medications.

**Table 2:** Distribution of the studied caregivers according to their socio-demographic and clinical data

Socio-demographic data	Total (N = 100)	
	**Frequency**	**%**
**Age (years)**		
♣ 20-	7	7
♣ 30-	30	30
♣ 40-	41	41
♣ 50–60	22	22
Min. – Max.	21–57	
Mean ± SD.	41.57 ± 2.78	
**Sex**		
♣ Female	83	83
♣ Male	17	17
**Marital status**		
♣ Married ♣ Single	63 29	63 29
♣ Divorced/ Separated/ Widowed	8	8
**level of education**		
♣ Read and write/ Basic education	10	10
♣ Secondary education ♣ University education ♣ Postgraduate education	19 51 20	19 51 20
**Relationship to the care receiver**		
♣ Daughter ♣ Son ♣ Daughter-in-law ♣ Grandchild ♣ Spouse	73 16 5 3 3	73 16 5 3 3
**Living with the care receiver**		
♣ Yes	67	67
♣ Yes, temporarily ♣ No	18 15	18 15
**Having children under the age of 18**		
♣ No	54	54
♣ Yes	46	46
**Working beside caregiving**		
♣ Not working	50	50
♣ Employee	50	50
**Income**		
♣ Enough	61	61
♣ Not enough	39	39
**Caregiving hours / day**		
♣ < 6	29	29
♣ 6-	23	23
♣ 12-	23	23
♣ 18–24	25	25
**Duration of acting as a caregiver**		
♣ Less than 6 months	10	10
♣ From 6 months to less than 1 year	12	12
♣ From 1–2 years	19	19
♣ More than 2 years	59	59
**Having someone help in caregiving**		
♣ No, I am the only caregiver	34	34
♣ Family member/friends	56	56
♣ Professional Care Providers	7	7
♣ Friends	3	3
**Suffering from chronic illness**		
♣ No	48	48
♣ One	25	25
♣ Two	16	16
♣ Three and more	11	11
**Consuming medications**		
♣ Yes ♣ No	55 45	55 45

Table 3 shows that the mean age of older adults with Alzheimer’s disease was 75.35 ± 6.514 years (range 60 to 89 years). The majority of adults with Alzheimer’s disease were female (80%), and 20% of them were males. Regarding the duration of being diagnosed with AD, 61% of older adults were diagnosed with AD for more than two years. Concerning the mobility status of older adults with AD, 38% of them moved without help, while 30% of them needed full assistance. Approximately three-quarters of the older adults with AD had chronic diseases (73%). Hypertension, diabetes mellitus, and heart diseases were the most prevalent chronic diseases and were reported by 52%, 33%, and 18% of the caregivers of older adults with AD, respectively. Finally, approximately 79% of the patients with AD took medications.

**Table 3:** Distribution of the Alzheimer’s older adults according to their socio-demographic and clinical data (Total N = 100)

Socio-demographic data	Frequency	%
**Age (years)**		
♣ 60-	5	5
♣ 65-	11	11
♣ 70-	26	26
♣ 75-	31	31
♣ 80 and more	27	27
Min. – Max.	60.0 − 89.0	
Mean ± SD.	75.35 ± 6.514	
**Sex**		
♣ Female	80	80
♣ Male	20	20
**Duration of being diagnosed with AD**		
♣ Less than 6 months	13	13
♣ 1 year	11	11
♣ 2 years	15	15
♣ More than 2 years	61	61
**Mobility of Alzheimer’s older adults**		
♣ Moves without help ♣ Moves with the help of a person ♣ Moves using assistive devices such as cane or walker ♣ Needs full assistance	38 21 11 30	38 21 11 30
**Medical history of chronic diseases**		
♣ No	27	27
♣ Yes #	73	73
♣ Hypertension ♣ Diabetes mellitus	52 33	52 33
♣ Heart disease	18	18
♣ Musculoskeletal diseases	14	14
♣ Respiratory diseases	12	12
♣ Renal impairment	11	11
♣ Parkinson’s disease	4	4
♣ Others	9	9
**Consuming medications**		
♣ Yes	79	79
♣ No	21	21

Table 4 shows that the mean knowledge level about Alzheimer’s disease of the studied caregivers was 13.02 ± 4.304 before the implementation of the program, and it increased to 27.43 ± 1.273 after the implementation of the program, and the difference was highly statistically significant (p = 0.000).

**Table 4:** Mean knowledge level of the studied caregivers, before and after the implementation of the study intervention

**Item**	Total (N = 100)		t	P
	**Pre-intervention**	**Post-intervention**	-30.707	0.000*
	**Mean ± SD**	**Mean ± SD**		
**Caregivers’ Alzheimer disease knowledge level**	13.02 ± 4.304	27.43 ± 1.273		

t = Paired samples t-test * Significant at p ≤ 0.05

Table 5 reveals that the mean stress level of the studied caregivers of older adults with AD was 27.50 ± 9.482 before the implementation of the study program, and it decreased to 18.92 ± 3.569 after the program implementation, with a highly statistically significant difference between both means of caregiver stress level (P = 0.000).

**Table 5:** Mean stress level of the studied caregivers, before and after the implementation of the study intervention

**Item**	Total (N = 100)		t	p
	**Pre-intervention**	**Post-intervention**	8.500	0.000*
	**Mean ± SD**	**Mean ± SD**		
**Caregivers’ stress level**	27.50 ± 9.482	18.92 ± 3.569		

t = Paired samples t-test * Significant at p ≤ 0.05

Table 6 illustrates that the mean psychological wellbeing score of the studied caregivers of older adults with AD was 35.96 ± 9.143 before the implementation of the study program, while it increased to 40.71 ± 4.785 after the implementation of the study program, with a highly statistically significant difference (P = 0.000).

**Table 6:** Mean psychological wellbeing of the studied caregivers, before and after the implementation of the study intervention

**Item**	Total (N = 100)		t	p
	**Pre-intervention**	**Post-intervention**	-4.763	0.000*
	**Mean ± SD**	**Mean ± SD**		
**Caregivers’ psychological wellbeing**	35.96 ± 9.143	40.71 ± 4.785		

t = Paired samples t-test * Significant at p ≤ 0.05

Table 7 shows that the effect size of the study program on the caregivers’ knowledge level was 1.107, which was a significantly large effect. Moreover, the study program revealed a large effect on the studied caregivers’ stress level (3.12). On the other hand, the study program had a large effect size on the studied caregivers’ psychological well-being (1.32).

**Table 7:** The intervention’s effect size on knowledge, stress level, and psychological wellbeing mean scores of caregivers of Alzheimer’s older adults

Items	Study Group (N = 100)		Mean Change	Effect size
	**Before**	**After**		
	Mean ± SD	Mean ± SD		
Caregivers’ Alzheimer disease knowledge level	13.02 ± 4.304	27.43 ± 1.273	-14.410	1.11
Caregivers’ stress level	27.50 ± 9.482	18.92 ± 3.569	8.580	3.12
Caregivers’ psychological wellbeing	35.96 ± 9.143	40.71 ± 4.785	-4.750	1.32

Effect size 0.0-0.2 Small effect 0.3–0.7 Medium effect ≥ 0.8 Large effect

## Discussion

AD is known as a family disease because the persistent anguish of seeing a loved one progressively deteriorates affects everyone. Therefore, a successful therapy needs to take into account the demands of the entire family. Caregivers are sometimes overlooked, disregarded, dismissed as inconsequential, or seen as an afterthought. The importance of caregiving cannot be overstated. Primary caregivers are just as vital as physicians and nursing personnel in the treatment of Alzheimer’s geriatric patients [48]. The successful treatment and rehabilitation of sick patients, particularly in cases of AD, depends largely on caregivers who bear the burden of day-to-day care of their loved ones. Therefore, this study aimed to determine the effect of a PFA program on stress levels and psychological well-being among caregivers of older adults with AD.

The present study showed that the majority of the caregivers were females, daughters, living with their patients in the same house, and they were looking after their Alzheimer’s patients for more than 2 years, and there was someone else helping them in caring. That was justified as in Arab Muslim society; there are religious and spiritual beliefs connected to their family beliefs of honoring, respecting, and taking care of their parents. These beliefs endorse the meaning of familism and the motivation to become a caregiver. Additionally, according to Egyptian culture, most families are extended families, and everyone must look after each other. These results are consistent with a study performed by **Cody et al., 2021**, who reported that the majority of informal caregivers were females and lived in the same household as their patients [48]. Another study performed on caregivers for Alzheimer’s patients reported that the prevalent caregivers of the patients were wives or daughters who cared for the patient at home [49]. The present result is also consistent with that of the study performed by **Cody et al., 2021**, who studied the burdens of the caregivers of Alzheimer’s patients and reported that most caregivers had been providing care for 3–5 years, followed by 6 + years [48].

The present study shows that almost half of the caregivers were not working, and more than one-third of them did not have enough income. In addition, approximately half of the studied caregivers of Alzheimer’s patients take from 12 to 24 h of caregiving daily. This can be explained by the fact that the burden of performing various tasks, such as feeding, protecting their patients, mobility, personal hygiene, and giving medications, affects all aspects of the caregiver’s life and takes most of the caregiver’s time. This is consistent with a study done by **Cheng, 2017**, who pointed out that the caregivers of Alzheimer’s geriatric patients were obliged to decrease the working hours to look after their relatives as well as daily life activity disruption [50]. In the present study, approximately two-thirds of the studied caregivers had university or postgraduate education. This is consistent with the results of a study performed by **Cody et al., 2021**, who found that the majority of years of caregiver formal education were college and postgraduate education [48]. In addition, **Zahed et al., 2020** studied 99 Alzheimer’s caregivers and found that approximately one-third of them were highly educated females [51].

The present study shows that more than half of the studied caregivers suffered from chronic illness and took some types of medications. A study by **Tulek et al., 2020** on burden, quality of life, and related factors in family caregivers of dementia patients in Turkey found that approximately 60% of the studied caregivers suffered from chronic illness [52]. Additionally, another study performed by **Mendez et al. (2021)** reported that approximately 47% of the caregivers of patients with dementia suffer from hypertension and approximately 20% have diabetes mellitus [53].

Regarding caregivers’ knowledge about AD, the present study showed that before the application of the study program, the level of knowledge about AD was low, as the mean score was 13.02. The key explanation of this result is that most of the caregivers were unable to obtain adequate and reliable information because they simply did not know what to ask for and whom to ask. This was also justified by a qualitative question asked by the authors in the present study about the caregivers’ needs, most of whom reported, “we need to know everything about AD, and how we can look after our relatives”. This finding is in line with that of a study performed by **Vara-García et al., (2021)**, who reported a lack of knowledge and information about AD among caregivers, and there is a need to increase their knowledge related to the identification of dementia symptoms and the progression of the disease through psychoeducational sessions for family caregivers [54].

After the application of the study program, the mean score of caregivers’ knowledge level about AD increased from 13.02 to 27.43, and the increase in knowledge level about AD was found to be highly statistically significant, with an effect size of 1.107. This could be attributed to the fact that the PFA program was prepared based on the caregivers’ needs and interests, so they complied with the program sessions. These results are consistent with those of a study performed by **Tomar et al., 2019**, who used a dementia first aid program for the family caregivers of patients with AD in Iran, and found a significant increase in the level of knowledge after the application of the program and that improvement was sustained for 6 months [55].

The level of stress experienced by the studied caregivers in the present study was moderate before applying PFA, as the mean stress level score was 27.5. The increase in the stress level among the studied caregivers can be explained by the fact that the majority of them are females, married, and have their own family to look after them. The majority of them suffered from chronic diseases that required treatment. In addition, a long duration of caregiving during the day with little support may be a factor. Furthermore, the majority of Alzheimer’s patients were more dependent on their caregivers in mobility and daily activities. They also suffered from chronic medical diseases and took medications for a long duration of being diagnosed with AD. All of these stressors and responsibilities make the term “sandwich generation” true for those caregivers [51]. The result of perceived stress among caregivers of patients with Alzheimer’s is consistent with **Zahed et al., 2020**, who reported that most of the caregivers suffer from a moderate level of stress, and the mean score was significantly higher in the female caregivers [51]. Another study performed by **Anand et al., 2016** revealed that perceived stress among caregivers of patients suffering from AD was three times higher than stress among caregivers of patients with chronic diseases [56].

After the application of the PFA in the present study, a significant decrement in the mean score of the studied caregivers’ perceived stress was observed, with a mean difference of 8.5 and a large effect size of 3.12. The decrement in the stress level among the studied caregivers can be explained by the fact that the mean focus of the PFA program is to provide emotional and practical support to the caregivers at the time of stress-related caregiving. In addition, the authors in the present study used stress-reduction techniques during and between session programs, such as relaxation techniques, deep breathing exercises, imagination, and sublimating hold destructive feelings, by using physical exercises.

The previous results were supported by a similar intervention performed by **Savundranayagam et al., 2011**, which used a psychoeducational program called “powerful tools of caregivers” to decrease stress burden among spouses with disabled patients. They pointed out that the caregiver spouse who participated in the program reported a significantly lower stress burden than those who did not participate [57]. Another study was performed by **Leszko, 2019**, using a psychoeducational and financial intervention to decrease the burden among caregivers of patients with Alzheimer’s. They pointed out that the program successfully decreased the caregivers’ stress not only for a short period but also for 6 months after the program was implemented [58]. A cohort study performed by **Lethin et al., 2017**, revealed that caregiver stress burden, quality of life, and neuropsychiatric symptoms in patients with dementia were associated with decreased psychological well-being. Eventually, all those factors mentioned in the previous study were included in the sessions of the present PFA program [59].

Concerning the effect of the PFA program on psychological wellbeing among caregivers of patients with AD, the present study showed that there was a significant increase in psychological wellbeing among caregivers after the application of the PFA program, with a mean difference of 4.75 and a large effect size of 1.32. A study by **Frias, Risco, & Zabalegui, 2020** contradicted the present study and found that applying psychoeducational intervention on the burden and emotional well-being among informal caregivers of people with dementia can lead to a slight insignificant improvement in psychological wellbeing among them because of low perceived social support [60].

The study findings support the study hypotheses, as the caregivers of older adults with AD experienced a decrement in stress levels and an improvement in their knowledge and psychological well-being after the application of the PFA program. This can be explained by the fact that the researchers acted as active listeners to the caregivers, carefully assessed their needs and concerns, and then tailored the nursing interventions and sessions according to those needs. PFA aided in establishing feelings of security, comfort, self-social efficacy, belongingness, and hopefulness. Therefore, PFA can be safely and effectively incorporated into standard nursing practice in the management of AD caregivers’ emotional stress, burden, anxiety, and feelings of burnout, which in turn improves the quality of care provided by caregivers to their older adults with AD.

### Limitations of the study

Despite the significant results of the present study, the lack of a control group hinders the validity of the results, which is the strongest limitation. The second limitation was the inability of the researchers to obtain the stage of AD from the caregivers because they did not know to specify at which stage their patients were. Moreover, the researchers used a virtual method of data collection, which hindered them from assessing the AD patients’ health status directly due to the COVID-19 pandemic.

## Conclusion

Based on the results of the current study, it can be concluded that, the knowledge of the caregivers of older adults with AD as well as their psychological well-being had been improved after the implementation of the PFA program than before it with a highly statistically significant effect. On the other hand, the stress level of the caregivers of older adults with AD was reduced after the implementation of the study program than before it, and the difference was highly statistically significant. Both study hypotheses are supported by the study results.

### Recommendation

- Future research should utilize a control group to further develop effective interventions to decrease stress levels and enhance psychological well-being among AD caregivers.
- Future research needs to be conducted face-to-face to compare the effects of PFA intervention on the stress level and psychological wellbeing among caregivers of AD patients with different stages of illness.

## Data Availability

Data will be available from the authors upon reasonable request.

## Abbreviations

PFA: Psychological first aid
AD: Alzheimer’s disease.
ADLs: Activities of Daily Living.
PWB: Psychological wellbeing.
PTSD: Posttraumatic stress disorder.
ADKS: Alzheimer Disease Knowledge Scale.
KCSS: Kingston Caregiver Stress Scale.
AIM: Authentic Identity Measures.
